# Regulating medical AI before midnight strikes: Addressing bias, data fidelity, and implementation challenges

**DOI:** 10.1371/journal.pdig.0000986

**Published:** 2025-08-18

**Authors:** Cameron J. Sabet, Ketan Tamirisa, Danielle Sara Bitterman, Leo Anthony Celi

**Affiliations:** 1 Department of Orthopedics, Georgetown University School of Medicine, Washington, DC, United States of America; 2 Department of Public Health, Washington University in St. Louis, St. Louis, Missouri, United States of America; 3 Department of Radiation Oncology, Massachusetts General Hospital, Boston, Massachusetts, United States of America; 4 Department of Medicine, Beth Israel Deaconess Medical Center, Boston, Massachusetts, United States of America,; 5 Division of Clinical Informatics, Massachusetts Institute of Technology, Cambridge, Massachusetts, United States of America; Emory University, UNITED STATES OF AMERICA

The potential of artificial intelligence (AI) in healthcare is truly enormous. However, despite extensive theoretical discussions about AI ethics, real-world regulatory implementation remains limited [1]. For instance, in May 2024, President Joe Biden gathered the CEOs of the leading four AI companies to discuss the responsibilities each holds in ethics and risk management [2]. Notably absent were ethicists and representatives of the everyday Americans most affected by these technologies. While AI excels in image analysis and predictive diagnostics, its progress must be matched with industry-regulator-ethicist discussions on biases, data quality, and decision-making transparency.

Firstly, AI’s foundation, data, is itself deeply flawed. De-identified electronic health record (EHR) data, commonly used to train AI systems, are often riddled with inconsistencies [3,4]. Thus, these datasets must be carefully curated. The National Clinical Cohort Collaborative (N3C), which harmonizes data from over 75 institutions, offers a valuable model for creating inclusive datasets [5]. Such networks should serve as templates for chronic diseases, rare diseases, and any populations underrepresented in the trials underpinning U.S. Food and Drug Administration (FDA) approvals. Similarly, the All of Us Research Program at the National Institutes of Health, which seeks to develop a nationwide database of 1 million people, reflecting a more diverse US population, represents progress but remains neither large nor fast enough [6]. To improve data collection, the US Congress can mandate a standardized national template for EHR interoperability [7]. Introducing this standard template for patient records across all hospitals nationwide can help expand the training datasets and ensure more standardized input of de-identified patient information to more accurately train AI algorithms.

AI systems trained on biased datasets risk exacerbating health disparities [8]. To counter this, AI systems should undergo bias audits using established open-source tools like IBM’s AI Fairness 360 [9]. More importantly, algorithmic decision-making in health care raises ethical questions about appropriate levels of accountability and consent due to the lack of explanations in AI responses. These considerations should remain central as startups automate decisions such as triage, which becomes critical during times of resource scarcity, as seen in the recent COVID-19 pandemic [10]. The confidentiality of health data is also a concern, as AI models often store inputs for training, personalization, or centralized processing. Health professionals need explainable outputs to make informed decisions, and patients deserve transparency regarding their individual treatment plans. Northeastern University has established an AI Ethics Board of 40 ethicists and community members to review AI initiatives and provide detailed implementation plans [11]. For-profit companies and hospitals like Massachusetts General Brigham or Tufts Medical Center can adopt similar models, acting like Institutional Review Boards (IRBs) to evaluate AI-based tools before implementation. These boards should incorporate diverse community members to ensure that the populations being treated feel adequately represented in the decisions behind these significant shifts in their care [12].

AI models can lead to serious privacy concerns due to their storage of input data for training, personalization, or central processing. Emerging privacy-preserving techniques such as federated learning and differential privacy show strong promise. Google’s Android and Apple’s iOS have implemented such privacy-preserving methods at a massive scale [13–16]. In Europe, the FeatureCloud project uses federated learning across institutions to develop models without compromising privacy [17]. US hospitals could adopt similar privacy-protecting collaborations.

AI implementation in medicine also depends on clinical readiness. Through its certification programmes, the American Board of Artificial Intelligence in Medicine (ABAIM) is already working to ensure that doctors, nurses, and other care specialists are prepared for the use of AI in medicine [18]. These initiatives should expand into medical curricula and patient education programs to equip patients for informed decisions about AI-driven care. Simultaneously, AI development teams should reflect the populations they serve, and ethnic, geographic, and socioeconomic diversity in design teams is critical to prevent blind spots and systemic bias. The American Medical Association’s External Equity & Innovation Advisory Group could join the Partnership on AI to bridge the gap and increase minority participation in healthcare AI [19].

Ethical development of AI necessitates robust governance. The FDA’s draft regulatory pathway for Artificial Intelligence and Machine Learning Software as a Medical Device (SaMD) Action Plan provides a useful starting point for other organizations to follow suit [20]. These AI guidelines exist within FDA partnerships, but agencies like HHS and CMS have yet to adopt similar regulations. To accelerate these developments, the HHS could partner with the FDA and extend core AI device principles to other applications in healthcare, including automated prior authorizations for Medicare Part C Advantage plans.

Post-deployment monitoring is also just as essential. Healthcare AI should adopt continuous audit mechanisms to detect and address failures in real-time by drawing inspiration from the Federal Aviation Administration’s black boxes, or the FDA’s Adverse Event Reporting System (FAERS) [21,22]. Without these, troubleshooting AI systems, especially in high-stakes settings, will be extremely difficult.

Causal inference offers another layer of reliability. Causal models can allow developers to move beyond correlation to understand cause–effect relationships, which are vital for specific treatment recommendations and triage. Columbia’s Causal AI Lab is one of many efforts pursuing this initiative, alongside Microsoft’s DoWhy program and MIT’s work in healthcare-specific causal frameworks [23–25]. Clinical centers, collaborating with academia, should integrate these tools and train clinicians to understand and interpret them. The NIH’s Bridge2AI program should prioritize structured datasets for causal analysis [26].

Effective implementation depends heavily on the political climate. Under the current administration, recent moves such as the National Institute of Standards and Technology (NIST) restricting terms such as “AI safety,” “responsible AI,” and “AI fairness” may lead to a growing aversion to ethical AI discourse [27]. With DEI principles currently under attack and ethics reframed as ideologically loaded, advancing these recommendations would likely require concerted, bottom-up efforts from health institutions, advocacy groups, and local governments alike.

AI in healthcare presents challenges, but progress can be made with realistic strategies, collaboration, and shared learning. While ongoing effort and investment are needed, a well-implemented approach can ensure AI benefits all stakeholders—most importantly, patients. The US could mirror the UK’s approach, where the National Health Services AI Laboratory has created frameworks to measure clinical, operational, and economic impacts [28]. With appropriate training, oversight, and governance, AI can improve rather than endanger health equity.
